# Surface proteins that promote adherence of *Staphylococcus aureus *to human desquamated nasal epithelial cells

**DOI:** 10.1186/1471-2180-9-22

**Published:** 2009-01-30

**Authors:** Rebecca M Corrigan, Helen Miajlovic, Timothy J Foster

**Affiliations:** 1 Microbiology Department, Moyne Institute of Preventive Medicine, Trinity College, Dublin 2, Ireland

## Abstract

**Background:**

The natural habitat of *Staphylococcus aureus *is the moist squamous epithelium in the anterior nares. About 20% of the human population carry *S. aureus *permanently in their noses and another 60% of individuals are intermittent carriers. The ability of *S. aureus *to colonize the nasal epithelium is in part due to expression of surface proteins clumping factor B (ClfB) and the iron-regulated surface determinant A (IsdA), which promote adhesion to desquamated epithelial cells present in the anterior part of the nasal vestibule. *S. aureus *strain Newman defective in IsdA and ClfB exhibited reduced but not completely defective adherence to squamous cells in indicating that other cell surface components might also contribute.

**Results:**

Surface proteins IsdA, ClfB, and the serine-aspartic acid repeat proteins SdrC, SdrD and SdrE were investigated to determine their contribution to the adherence of *S. aureus *to desquamated nasal epithelial cells. This was achieved by expression of ClfB, IsdA, SdrC, SdrD and SdrE on the surface of the surrogate Gram-positive host *Lactococcus lactis *and by isolating mutants of *S. aureus *Newman defective in one or more factor. The level of adherence of strains to squamous cells isolated from the nares of volunteers was measured. Results consistently showed that ClfB, IsdA, SdrC and SdrD each contributed to the ability of *S. aureus *to adhere to squamous cells. A mutant lacking all four proteins was completely defective in adherence.

**Conclusion:**

The ability of *S. aureus *Newman to adhere to desquamated nasal epithelial cells is multifactorial and involves SdrD and SdrC as well as ClfB and IsdA.

## Background

*Staphylococcus aureus *causes community-acquired and nosocomial infections. Although multiple body sites such as the axilla and the perineum can be colonized, the most frequent site of carriage is the moist squamous epithelium of the anterior nares. About 20% of the human population carry *S. aureus *permanently in their noses and another 60% of individuals are intermittent carriers [1]. The reasons for the variable tropism of *S. aureus *for the human nares are unclear. Higher carriage rates occur in white people [2], in men [2], in certain age groups [3] and in dialysis [4], diabetic [5] and AIDS patients [6]. Infection rates are higher in carriers than in non-carriers and invasive disease is often caused by a patients' carried strain [7]. However when infected, carriers suffer significantly fewer fatalities, suggesting that carriage stimulates a degree of protective immunity [8].

It has been suggested that the ability of *S. aureus *to adhere to human desquamated nasal epithelial cells is an important factor in determining nasal colonization [9]. Both clumping factor B (ClfB) and iron regulated surface determinant protein A (IsdA) are expressed on the bacterial cell surface and promote adhesion to desquamated epithelial cells *in vitro *and colonization of the nares of rodents in *in vivo *models [10,11], and in the case of ClfB [12], humans. Protection against colonization was elicited by active immunization of rodents with recombinant ClfB or IsdA, and in the case of ClfB, with a function-blocking monoclonal antibody.

The surface protein SasG can also promote adhesion to desquamated nasal epithelial cells *in vitro *[13,14]. However SasG is not expressed by many strains including Newman [14]. A mutant of *S. aureus *strain Newman defective in IsdA and ClfB had reduced adherence to squamous cells but still bound at about 40% of the level of the wild-type [10]. Since SasG is not expressed by strain Newman [14], other cell surface components are likely to be involved. It had been noted that the serine-aspartic acid repeat proteins SdrC and SdrD can also promote adhesion to squamous cells [11], although this has never been examined in detail. In this paper the role of surface proteins IsdA, ClfB, SdrC and SdrD in adhesion to desquamated cell has been systematically analyzed in order to determine the contribution of each under the same conditions. This was achieved by expression of ClfB, IsdA, SdrC and SdrD on the surface of the Gram-positive surrogate host *Lactococcus lactis *and by testing single and combined mutants of *S. aureus *Newman.

## Results

### Expression of IsdA, ClfB, SdrC and SdrD by the surrogate host *L. lactis*

**Figure 1:**
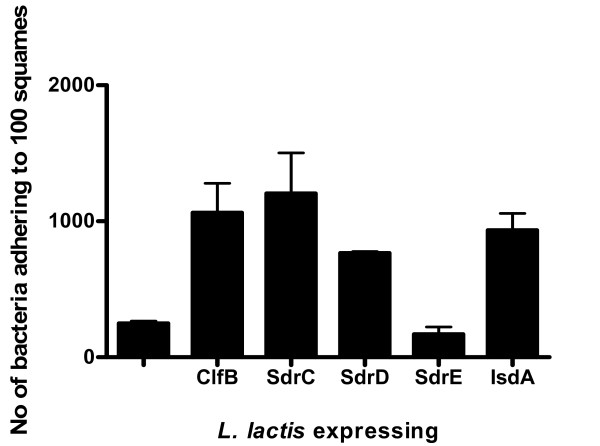
**Adherence of *L. lactis *expressing different surface proteins to desquamated nasal epithelial cells**. *L. lactis *(pKS80), *L. lactis *(pKS80*clfB*^+^), *L. lactis *(pKS80*sdrC*^+^), *L. lactis *(pKS80*sdrD*^+^), *L. lactis *(pKS80*sdrE*^+^) and *L. lactis *(pKS80*isdA*^+^) grown to stationary phase were tested for their ability to bind to human desquamated epithelial cells. Counts represent the number of bacterial cells adhering to 100 squamous cells. Results are expressed as the mean of triplicate experiments +/- standard deviations.

To determine the ability of the IsdA, ClfB, SdrC, SdrD and SdrE proteins to promote adhesion to human desquamated nasal epithelial cells, *L. lactis *cells expressing each protein [9] were incubated with squamous cells from the anterior nares of healthy volunteers. *L. lactis *containing the empty vector pKS80 adhered poorly (Figure 1). *L. lactis *expressing SdrE was not significantly different to *L. lactis *carrying pKS80 (*P *= 0.2055; Figure 1) indicating that this protein cannot promote adhesion to squamous cells. In contrast, a significant increase in adherence to squamous cells was observed when *L. lactis *cells expressed SdrC, SdrD, ClfB or IsdA (*P *values of 0.0339, SdrC; *P *= 0.0003, SdrD; *P *= 0.0396, ClfB and *P *= 0.0178, IsdA; Figure 1) showing that each of these proteins can promote adhesion when expressed on the surface of a Gram positive coccus. It was shown previously that ClfA expressed by *L. lactis *did not promote adhesion [15].

### Adherence of *S. aureus *mutants to desquamated nasal epithelial cells

**Figure 2:**
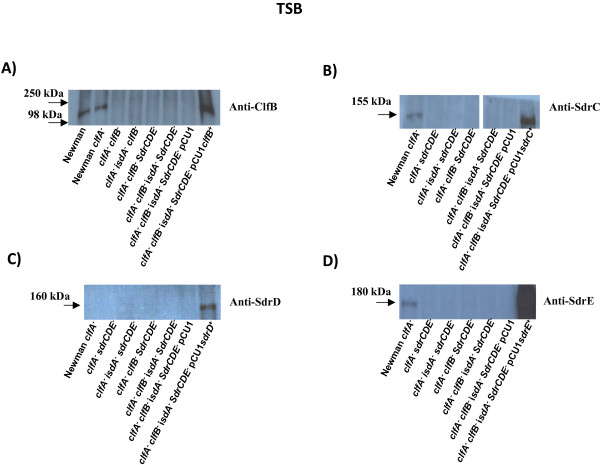
**Western immunoblot to detect expression of ClfB, SdrC, SdrD and SdrE**. A-D. Cell wall associated proteins from strains Newman, Newman *clfA*, Newman *clfA clfB*, Newman *clfA isdA clfB*, Newman *clfA clfB sdrCDE*, Newman *clfA isdA clfB sdrCDE*, Newman *clfA sdrCDE*, Newman *clfA isdA sdrCDE *and Newman *clfA isdA clfB sdrCDE *(pCU1) and (A) Newman *clfA isdA clfB sdrCDE *(pCU1*clfB*^+^), (B) Newman *clfA isdA clfB sdrCDE *(pCU1*sdrC*^+^), (C) Newman *clfA isdA clfB sdrCDE *(pCU1*sdrD*^+^), (D) Newman *clfA isdA clfB sdrCDE *(pCU1*sdrE*^+^) were grown to the exponential phase in TSB, cell wall associated proteins were solubilised by lysostaphin and run on 7.5% SDS-PAGE gels. Western immunoblotting was performed with (A) rabbit anti-ClfB antibodies, (B) rabbit anti-SdrC antibodies, (C) rabbit anti-SdrD antibodies and (D) rabbit anti-SdrE antibodies and subsequently with HRP-conjugated protein A-peroxidase.

In order to investigate the role of surface proteins in promoting adherence of *S. aureus *to desquamated nasal epithelial cells a set of isogenic mutants was constructed and compared. Strain Newman defective in *clfA *was used as the starting point in the strain construction but this mutation had no bearing on adhesion since ClfA is known not to promote adhesion to squamous cells [9]. Each strain was examined by Western immunoblotting in order to show that the relevant proteins were missing in the mutants and that the remaining proteins were expressed at the same level as in the wild type. Newman *clfA *grown to exponential phase in TSB expressed ClfB, SdrC and SdrE but not SdrD (Figure 2). Since bacteria were grown in TSB they did not express Isd proteins. Introduction of the multicopy shuttle plasmid pCU1 bearing the *clfB*, *sdrC *or *sdrE *genes resulted in expression of proteins at levels equivalent to or higher than the wild-type. In the case of SdrD expression was not seen in the wild-type strain and was only detected when the pCU1*sdrD*^+ ^plasmid was present (Figure 2C). This may be due to amplification of low level expression under these growth conditions due to a gene dosage affect by a multicopy plasmid.

**Figure 3:**
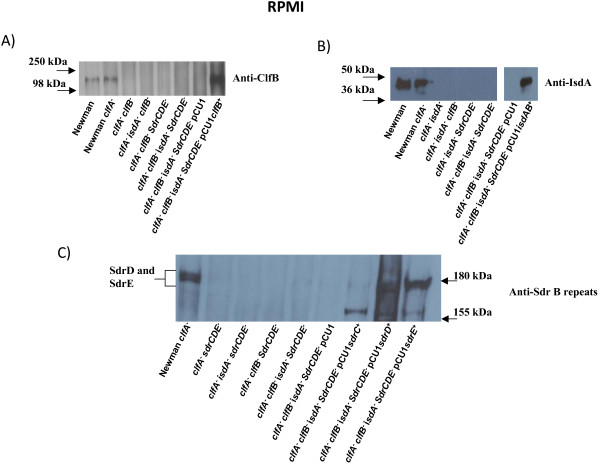
**Western immunoblot to detect expression of surface protein under iron-limiting conditions**. Bacteria were grown to stationary phase in RPMI. Cell wall associated proteins were solubilized with lysostaphin and separated on a 7.5% SDS-PAGE gel and detected with rabbit antibodies followed by HRP-conjugated protein A-peroxidase. (A). Newman wild-type, Newman *clfA*, Newman *clfA clfB*, Newman *clfA isdA clfB*, Newman *clfA clfB sdrCDE*, Newman *clfA isdA clfB sdrCDE*, Newman *clfA isdA clfB sdrCDE *(pCU1) and Newman *clfA isdA clfB sdrCDE *(pCU1*clfB*^+^). (B). Newman wild type, Newman *clfA*, Newman *clfA isdA*, Newman *clfA isdA clfB*, Newman *clfA isdA sdrCDE*, Newman *clfA isdA clfB sdrCDE*, Newman *clfA isdA clfB sdrCDE *(pCU1) and Newman *clfA isdA clfB sdrCDE *(pCU1*isdAB*^+^). (C). Newman *clfA*, Newman *clfA sdrCDE*, Newman *clfA isdA sdrCDE*, Newman *clfA clfB sdrCDE*, Newman *clfA isdA clfB sdrCDE*, Newman *clfA isdA clfB sdrCDE *(pCU1), Newman *clfA isdA clfB sdrCDE *(pCU1*sdrC*^+^), Newman *clfA isdA clfB sdrCDE *(pCU1*sdrD*^+^) and Newman *clfA isdA clfB sdrCDE *(pCU1*sdrE*^+^). The primary antibodies used were (A) rabbit anti-ClfB (B) rabbit anti-IsdA and (C) rabbit anti-SdrD B repeats.

Bacteria were also grown to stationary phase in RPMI. The wild-type strain expressed ClfB, IsdA, SdrD and SdrE, but not SdrC at levels that were detectable by Western immunoblotting (Figure 3). The Sdr proteins were detected with antibodies that recognized the conserved B domains (Figure 3C) and specific anti-A domain antibodies (not shown). Complementation of the mutant strain lacking these surface proteins with pCU1*clfB*^+^, pCU *isdAB*^+^, pCU1*sdrD*^+ ^or pCU1*sdrE*^+ ^resulted in restoration of expression of the appropriate protein at levels similar to (IsdA) or higher than wild-type (ClfB, SdrD, SdrE). In the case of pCU1*sdrC*^+ ^low level expression was achieved.

**Figure 4:**
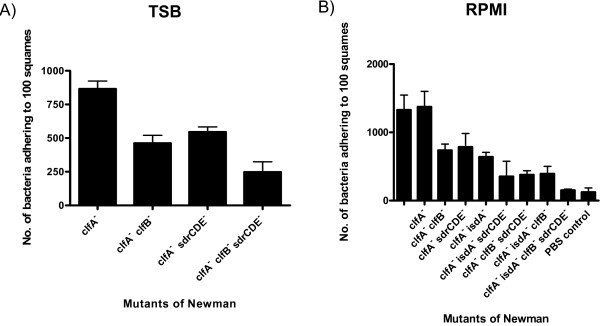
**Adherence of Newman mutants to desquamated nasal epithelial cells**. The ability of (A) Newman *clfA*, Newman *clfA clfB*, Newman *clfA sdrCDE *and Newman *clfA clfB sdrCDE *grown to exponential phase in TSB and (B) Newman, Newman *clfA*, Newman *clfA clfB*, Newman *clfA sdrCDE*, Newman *clfA isdA*, Newman *clfA isdA sdrCDE*, Newman *clfA clfB sdrCDE*, Newman *clfA isdA clfB*, and Newman *clfA isdA clfB sdrCDE *grown to stationary phase in RPMI to adhere to desquamated human nasal epithelial cells was measured. The tenth track is a control without *S. aureus *showing background due to adherent bacteria from the donor. Counts represent the number of bacterial cells adhering to 100 squamous cells. Results are expressed as the mean of triplicate experiments +/- standard deviations.

With Newman *clfA *grown in TSB approximately 800 bacteria adhered per 100 squamous cells (Figure 4A). The level of adhesion was reduced to ca 500 bacteria per 100 squamous cells when either ClfB or a combination of SdrC, SdrD and SdrE proteins were missing (Figure 4A, *P *= 0.0392, ClfB; *P *= 0.0441, SdrCDE). Adherence was even lower when the *clfB *and *sdrCDE *mutations were combined (Figure 4A, *P *= 0.0233 for Newman *clfA clfB sdrCDE *compared to Newman *clfA*). As only SdrC and SdrE were expressed under these conditions (Figure 2) and as experiments with *L. lactis *(pKS80*sdrE*^+^) indicated that SdrE did not promote adhesion to squamous cells (Figure 1), it is likely that the decrease observed by disrupting the *sdrCDE *genes is due to the loss of SdrC.

In order to determine the role of IsdA in adherence, mutants were grown to stationary phase in the iron limited medium RPMI and tested for adhesion to squamous cells. Newman wild-type and Newman *clfA *adhered at similar levels of ca 1300 bacteria per 100 squamous cells (Figure 4B). This confirms that ClfA does not promote adhesion to squamous cells. Disruption of ClfB, IsdA or SdrCDE in the *clfA *mutant host each caused a drop in adherence to ca 800 bacteria per 100 squamous cells (Figure 4B). The decrease was statistically significant for IsdA (*P *= 0.0389, compared to Newman *clfA*) but not for ClfB or SdrCDE (*P *= 0.0662 and 0.1852, respectively compared to Newman *clfA*). Combining the *isdA *and *sdrCDE *mutations, the *clfB *and *sdrCDE *mutations or the *isdA *and *clfB *mutations decreased adherence further (Figure 4B, *P *= 0.0352, 0.0135 and 0.0183, respectively compared to Newman *clfA*). Finally when a mutant lacking ClfA, ClfB, IsdA and SdrCDE was tested, only 200 bacteria adhered per 100 squamous cells. Of the Sdr proteins only SdrD and SdrE were expressed by Newman growing in RPMI (Figure 3) (IsdA and ClfB are also expressed under these conditions [12,15]) and as SdrE does not promote adhesion it can be concluded that the decrease associated with the deletion of *sdrCDE *was due to the loss of the SdrD protein. In conclusion, these results are consistent with the data obtained with *L. lactis *and demonstrate a similar role for ClfB, IsdA, SdrC and SdrD in adhesion to squamous cells.

### Complementation

**Figure 5:**
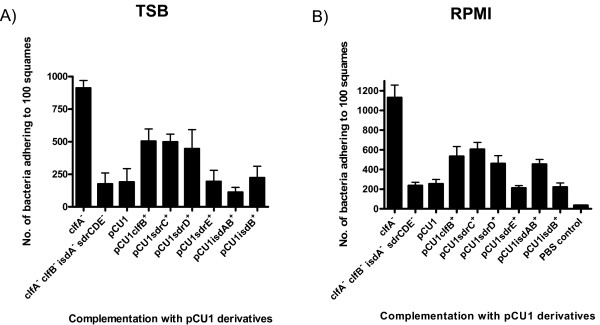
**Adherence of *S. aureus *Newman complemented mutants grown in TSB and iron restricted conditions to desquamated nasal epithelial cells**. The ability of mutants of strain Newman carrying complementing pCU1 plasmids carrying surface protein genes to adhere to desquamated nasal epithelial cells was tested. Strains Newman *clfA*, Newman *clfA isdA clfB sdrCDE*, Newman *clfA isdA clfB sdrCDE *(pCU1), Newman *clfA isdA clfB sdrCDE *(pCU1*clfB*^+^), Newman *clfA isdA clfB sdrCDE *(pCU1*sdrC*^+^), Newman *clfA isdA clfB sdrCDE *(pCU1*sdrD*^+^), Newman *clfA isdA clfB sdrCDE *(pCU1*sdrE*^+^),) Newman *clfA isdA clfB sdrCDE *(pCU1*isdAB*^+^) and Newman *clfA isdA clfB sdrCDE *(pCU1*isdB*^+^) grown to the exponential phase in (A) TSB and to the stationary phase in (B) RPMI were tested for adherence. Counts represent the number of bacterial cells adhering to 100 squamous cells. Results are expressed as the mean of triplicate experiments +/- standard deviations.

To confirm the roles of surface proteins in *S. aureus *deduced from the analysis of mutants, a strain of Newman that was defective in all four adherent surface proteins (in addition to ClfA and SdrE) was complemented by introducing multicopy shuttle plasmids expressing ClfB, SdrC, SdrD, SdrE, IsdAIsdB and IsdB. When bacteria were grown to exponential phase in TSB, ClfB, SdrC and SdrD each promoted increased adhesion to squamous cells above the background level (Figure 5A; *P *= 0.0554, ClfB; *P *= 0.0282, SdrC; *P *= 0.0449, SdrD; *P *= 0.8803, SdrE; *P *= 0.533, IsdA). The differences were statistically significant for SdrC and SdrD, but not for ClfB. Expression of SdrE did not promote adhesion which is consistent with results described above. The Isd proteins were not expressed in TSB-grown bacteria.

When the strains were grown under iron restricted conditions in RPMI, complementation with ClfB, IsdA, SdrC or SdrD each promoted adhesion (Figure 5B, *P *= 0.029, ClfB; *P *= 0.0536, SdrC; *P *= 0.0908, SdrD; *P *= 0.0384, IsdA). The conclusion about IsdA was drawn by comparing the level of adhesion promoted by the plasmid expressing both IsdA and IsdB with that expressing IsdB alone. Attempts to express IsdA alone in pCU1 were unsuccessful. These results were statistically significant except for those involving SdrC and SdrD. Expression of SdrE did not promote adhesion (Figure 5B). These results confirm that ClfB, IsdA, SdrC and SdrD are all important in adherence of *S. aureus *to desquamated nasal epithelial cells under growth conditions that pertain *in vivo*.

## Discussion

*S. aureus *is a commensal of the moist squamous epithelium of the anterior nares of a significant proportion of the population. Colonization is a known risk factor for the development of staphylococcal infections in the community and hospital. The causes of intermittent and persistent carriage are believed to be different. Persistent carriers are often colonised by a single strain of *S. aureus *over a long period of time, while intermittent carriers tend to carry different strains for briefer time periods [16,17]. Persistent carriers also carry a higher load of bacteria in the nares than intermittent carriers [18,19]. When volunteers were decolonized and were then inoculated with a mixture of *S. aureus *strains non-carriers eliminated the bacteria, whereas persistent carriers selected their original *S. aureus *colonizing strain from the mixture [20]. These results indicate that certain host characteristics determine carriage status in particular individuals. This view is supported by the fact that certain age or ethnic groups seem to be predisposed to carriage [2,3]. One determinant of varying patterns of nasal carriage may be differing expression levels of ligands for *S. aureus *on the surface of desquamated nasal epithelial cells.

In this study we used three donors to provide the desquamated nasal epithelial cells for adhesion experiments. They were selected because their cells supported a consistent level of adhesion. It has been noted that cells from different donors can provide widely variable levels of adhesion [21]. The reason for this is not known. One possibility is different levels of expression of the ligands responsible for adherence promoted by one or more of the *S. aureus *surface proteins. It is imperative to perform a detailed comparative study of the ability of the surface proteins described here to support adhesion of bacteria to squamous cells from donors who are persistent carriers and those who are non-carriers. This could contribute to the knowledge of the contribution of host factors to carriage.

Surface proteins ClfB and IsdA have previously been shown to promote adhesion to squamous epithelial cells [9,15] and are required for colonization of the nares of rodents [11,15]. Both ClfB and IsdA have been shown to bind to proteins present in the envelope of cornified squamous epithelial cells. IsdA and ClfB both bind to cytokeratin 10 and loricrin [22] (Clarke, S. Walsh, E. J. Andre, G. Dufrene, Y. Foster, T. J. Foster, S. J. manuscript submitted). Loricrin accounts for 70 – 85% of the cornified envelope [23-25]. It is possible that differences in the level of expression of these proteins could contribute to the variation in carriage of *S. aureus *in the nares.

To investigate the contribution of each of five surface proteins (IsdA, ClfB, SdrC, SdrD and SdrE) to squamous cell adhesion, the proteins were expressed from the surrogate host *L. lactis*. Expression of IsdA, ClfB, SdrC and SdrD each resulted in increased adherence. Gene disruption and complementation experiments in *S. aureus *also showed a role for IsdA, ClfB, SdrC and SdrD in adhesion. SdrE did not promote adhesion by either *L. lactis *or *S. aureus*. Schaffer *et al *2006 investigated whether SdrC or SdrD had a role in colonization of the nares in a mouse model. Mutants defective in SdrC or SdrD colonized mice to the same extent as the wild-type indicating that these proteins do not play a role colonization of the nares of mice [11]. However, this does not necessarily mean that SdrC and SdrD have no role to play in colonization of the human nares.

Adherence to desquamated epithelial cells from the anterior nares is clearly multifactorial. When expression of IsdA, ClfB, SdrC and SdrD was disrupted in strain Newman the level of adherence was reduced to background. This does not support a role for wall teichoic acid (WTA) in promoting adhesion to squamous cells [21,26]. SasG did not play a role in adherence of Newman to squamous cells because this protein was not expressed detectably by this strain despite the intact *sasG *gene being present [14]. SasG might play a role in clinical isolates where expression occurs at higher levels.

It has been reported that WTA plays a prominent part in nasal colonization of the cotton rat model [26]. The authors also demonstrated that teichoic acid promoted bacterial adhesion to normal epithelial cells. However the WTA apparently does not contribute to bacterial adhesion to desquamated nasal cells epithelial cells [21]. This is consistent with the data reported here which indicates that only surface proteins are required for adhesion to squames. A multiple mutant defective in ClfB, SdrC, SdrD and IsdA did not adhere. If WTA contributed to adherence the multiple mutant would still have bound above background levels. Thus colonization of the cotton rat requires both WTA [26] and surface proteins [15] albeit at different stages in the process [21] and in different parts of the nares.

## Conclusion

Eradication of carriage of *S. aureus *has been shown to reduce infection rates in dialysis, diabetic and AIDS patients [4-6]. Vaccination with IsdA and ClfB was effective in reducing *S. aureus *carriage in animal models [11,15]. It has been suggested that immune responses in part determine the ability of humans to carry *S. aureus *in the nares. This study has confirmed the role of ClfB and IsdA in adhesion to desquamated nasal epithelial cells and has revealed important roles for SdrC and SdrD. Vaccination against two or more of these surface proteins could provide significant reduction in carriage and could potentially reduce the rate of infection and dissemination.

## Methods

### Growth conditions

*Escherichia coli *strains were grown on Luria (L) agar or in L-broth (Difco). *S. aureus *strains were grown on tryptic soy agar (TSA; Oxoid), tryptic soy broth (TSB) or RPMI 1640 (Sigma). Cultures were grown in an orbital shaker at 200 rpm at 37°C. RPMI cultures were subcultured into fresh broth and grown for a further 15 h before harvesting. *L. lactis *strains were cultured in M17 medium (Difco) containing 0.5% (v/v) glucose without shaking at 30°C. Antibiotics (Sigma) were added when needed as follows: ampicillin (100 μg ml^-1^), erythromycin (10 μg ml^-1^), chloramphenicol (10 μg ml^-1^) or tetracycline (2 μg ml^-1^).

### Bacterial strains

The wild-type strain *S. aureus *strain Newman (10) and Newman *isdA *(RC107 Δ*isdA *[27]) were subjected to allele replacement mutagenesis with the temperature sensitive plasmid pJH1 [28] forming strains DU5999 *clfA5 *[28] and DU6020 *clfA5 isdA*. The *clfB*::Em^r ^mutation [29] and the Δ*sdrCDE*::Tc^r ^mutation [22] were introduced by transduction using phage 85 [30] in order to construct the following mutants of Newman: DU6000 *clfA5 clfB*::Em^r ^[28], DU6021 *clfA5 *Δ*sdrCDE*::Tc^r^, DU6001 *clfA5 clfB*::Em^r ^Δ*sdrCDE*::Tc^r ^[28], DU6022 *clfA5 isdA *Δ*sdrCDE*::Tc^r^, DU6023 *clfA5 isdA clfB*::Em^r ^Δ*sdrCDE*::Tc^r^. For each transductant the mutations were verified by PCR and Southern blotting.

Complementation analysis was performed by transferring into DU6023 *clfA5 isdA clfB*::Em^r ^Δ*sdrCDE*::Tc^r ^plasmid pCU1 containing the full length structural genes for *S. aureus *surface proteins as follows: pCU1*sdrC*^+^, pCU1*sdrD*^+^, pCU1*sdrE*^+^, pCU1*clfB*^+ ^[31], pCU1*isdAisdB*^+ ^and pCU1*isdB*^+^. The plasmids were maintained by selecting resistance to chloramphenicol (10 μg ml^-1^). In each case the gene was amplified from genomic DNA of strain Newman to include the promoter region and the downstream transcription terminator. In the case of isd proteins both the closely linked *isdA *and *isdB *genes and their cognate promoters were cloned together. The primers are described below.

Expression of surface proteins in *L. lactis *MG1363 [32] was achieved by cloning open reading frames from Newman genomic DNA in-frame into the expression vector pKS80 [33] forming pKS80*sdrC*^+ ^(25), pKS80*sdrD*^+ ^(25), pKS80*sdrE*^+ ^(25), pKS80*clfB*^+ ^(25) and pKS80*isdA*^+ ^(this study). Plasmid transformants were selected and maintained in M17 medium containing erythromycin.

### Molecular techniques

Standard procedures were used [34]. Restriction enzymes and ligase (New England Biolabs or Roche) were used according to manufacturer's protocol, as was Pfu DNA polymerase (Roche). Oligonucleotides were purchased from Sigma-Genosys.

### Plasmid and strain construction

Primers pCU1*sdrC*F (5'-CGGGGATCCAAGCTTAGATTAAAAGTGAG-'3) and pCU1*sdrC*R (5'-GCTCTAGACTGGGAATTTCTAAACAG-'3), pCU1*sdrD*F (5'-CGGGGATCCTTCTGTTTAGAAATTCCCAG-'3) and pCU1*sdrD*R (5'-GCTCTAGACCAGGCCTCACGGAC-'3) and pCU1*sdrE*F (5'-CCGGATCCGTAGAAACGAATAAGAAAAAGC-'3) and pCU1*sdrE*R (5'-GCTCTAGAGTAATTCATATTATCGCCTC-'3) which all incorporate a 5' *Bam*HI and '3 *Xba*I site, respectively, were used to amplify the *sdrC*, *sdrD *and *sdrE *genes, respectively, from strain Newman genomic DNA. The DNA containing the *sdrC*, *sdrD *and *sdrE *genes were digested with *Bam*HI and *Xba*I and cloned between the *Bam*HI and *Xba*I sites of plasmid pCU1.

Primers pCU1*isdB*F (5'-CAGCTGCAGCCTATGTCATAGATATTTCATAATC-'3) and pCU1*isdB*R (5'-CAGGAGCTCAGAGATTCTAAACGTATTCGTAAG-'3) which incorporate a 5' *Pst*I and 3' *Xba*I site, respectively, were used to amplify the *isdB *coding sequence including the upstream promoter and Fur consensus sequence from strain Newman genomic DNA. The *isdB *coding sequence is located 203 bp downstream of the *isdA *coding sequence on the *S. aureus *chromosome. Primers pCU1*isdA*F (5'-CAGCTGCAGACATAATCCTCCTTTTTATGATTG-'3) and pCU1*isdB*R (5'-CAGGAGCTCAGAGATTCTAAACGTATTCGTAAG-'3) were used to amplify the *isdA *and *isdB *coding sequence including the upstream promoter and Fur consensus sequence of both genes. The 2.3 kb *isdB *and 3.6 kb *isdAB *coding sequences were digested with *Pst*I and *Xba*I and cloned between the *Pst*I and *Xba*I sites of plasmid pCU1. Plasmids pCU1*isdB*^+ ^and pCU1*isdAB*^+ ^were sequenced and screened by restriction mapping.

Plasmids pCU1, pCU1*clfB*^+^, pCU1*sdrC*^+^, pCU1*sdrD*^+^, pCU1*sdrE*^+^, pCU1*isdB*^+ ^and pCU1*isdAB*^+ ^were transformed into *S. aureus *RN4220 and transduced into strain Newman *clfA clfB isdA sdrCDE *selecting for chloramphenicol resistance.

Primers FpKisdA (5'-CGCTGATCAAACATTATTTAAACAGTAAGTATC-'3) and RpKisdA (5'-CGCTGATCATTATTTAGATTCTTTTCTTTTGA-'3) which incorporate a 5' and a 3' *Bcl*I site, respectively, were used to amplify the *isdA *coding sequence from genomic DNA. The PCR product was digested with *Bcl*I and cloned into *Bcl*I digested pKS80. This resulted in the open reading frame of *isdA *being fused to the ATG codon of the expression cassette to optimize translation and created the plasmid pKS80*isdA*^+^. The plasmid was sequenced, screened by restriction mapping and electroporated into competent *L. lactis *strain MG1363.

### Western immunoblot analysis

Cell wall-associated proteins of *S. aureus *and *L. lactis *were prepared as previously described [35,22]. For *S. aureus *exponential phase cultures were grown to an OD_600 _of 0.6. Stationary phase cultures were grown for 16 – 24 h. Cells were harvested, washed in PBS and resuspended to an OD_600 _of 1 in lysis buffer (50 mM Tris/HCl, 20 mM MgCl_2_, pH 7.5) supplemented with 30% (w/v) raffinose and 40 μl ml^-1 ^protease inhibitors (Roche). Cell wall proteins were solubilized by incubation with lysostaphin (200 μgml^-1^) for 10 minutes at 37°C. Cell wall fractions were separated on 7.5% (w/v) polyacrylamide gels, electrophoretically transferred onto PVDF membranes (Roche), blocked in 10% (w/v) skimmed milk (Marvel) and probed with anti-ClfB antibodies (1:5,000; [31], anti-IsdA antibodies (1:2,000; a gift from Prof. P. Speziale, Department of Biochemistry, University of Pavia, Pavia, Italy) and anti-SdrC, anti-SdrD, anti-SdrE or anti-Sdr region B antibodies (1:2,000) [22]. The specificity of each antibody is indicated by the fact that no immnocrossreactive bands appeared in mutant strains lacking the relevant antigen. Membranes were washed three times with gentle agitation for 15 min in TS-Tween (10 mM Tris-HCl pH 7.4, 150 mM NaCl, 0.05% (v/v) Tween 20 (Sigma)). Bound antibodies were detected using horseradish peroxidase-conjugated protein A-peroxidase (1:500; Sigma). Proteins were visualised using the LumiGLO™ Reagent and peroxide detection system (Cell Signalling Technology^®^). Membranes were detected using Kodak X-ray film. The exposed films were fixed and developed using a Kodak X-OMAT 1000 Processor developing machine.

### Bacterial adherence to desquamated epithelial cells

Bacterial adherence assays were performed as previously described [13]. Briefly desquamated nasal epithelial cells were harvested from three healthy donors by vigorous swabbing of the anterior nares. One donor was a carrier of *S. aureus *while the other two were not. After washing in PBS, cells were adjusted to 1 × 10^5^cell ml^-1^. Bacterial cells were washed with PBS and adjusted to 1 × 10^9^cells ml^-1^. 100 μl volumes of bacterial and epithelial cells were mixed and incubated at 37°C for 1 h with occasional shaking before being captured on 12 μm isopore polycarbonate filters, washed with PBS, fixed and stained with 5% (w/v) crystal violet. The filters were mounted onto glass slides. The number of bacteria per 100 squames was counted using light microscopy.

### Statisical Analysis

Statistical analyses were determined by the Student t-test, using the online GraphPad software. Differences were considered significant if *p *values were less than 0.05.

## Authors' contributions

RMC carried out strain construction, performed Western immunoblotting, all squamous cell adhesion assays and drafted the manuscript. HM constructed five plasmids/strains for this study and helped to draft the manuscript and TJF conceived and coordinated the study, and helped to draft the manuscript. All authors read and approved the final manuscript.
